# Crystal structure of 2,2′-({[2-(trityl­sulfan­yl)benz­yl]azane­diyl}bis­(ethane-2,1-di­yl))bis­(isoindoline-1,3-dione)

**DOI:** 10.1107/S1600536814015554

**Published:** 2014-08-01

**Authors:** Ulrich Flörke, Adam Neuba, Jochen Ortmeyer, Gerald Henkel

**Affiliations:** a Universität Paderborn, Warburger Strasse 100, 33098 Paderborn, Germany

**Keywords:** crystal structure, tripodal ligands, phthalimide, hydrogen bonding

## Abstract

In the structure of the title compound, C_46_H_37_N_3_O_4_S, the planes of the two isoindoline units make a dihedral angle of 77.86 (3)°. The dihedral angles between the benzyl plane and the isoindoline units are 79.56 (4) and 3.74 (9)°. The geometry at the S atom shows a short [1.7748 (17) Å] S—C_benz­yl_ and a long [1.8820 (15) Å] S—C_trit­yl_ bond and the C—S—C angle is 108.40 (7)°. N—C bond lengths around the azane N atom are in the range 1.454 (2)–1.463 (2) Å. he crystal packing exhibts two rather ‘non-classical’ C—H⋯O hydrogen bonds that result in stacking of the molecules along the *a* as well as the *b* axis and give rise to columnar sub-structures.

## Related literature   

For related mol­ecular structures and bonding geometries, see: Barrett *et al.* (1995 ▶); Howell *et al.* (2003 ▶); Latxague *et al.* (2009 ▶) and Qi *et al.* (2009 ▶). For the modelling of the active center of the peptidglycine-α-hy­droxy­lating monooxygenase, see: Hoppe *et al.* (2013 ▶); Neuba (2009 ▶). For inter­mediate steps of the synthesis, see: Formica *et al.* (2002 ▶) and Sagrera & Seoane (2009 ▶).
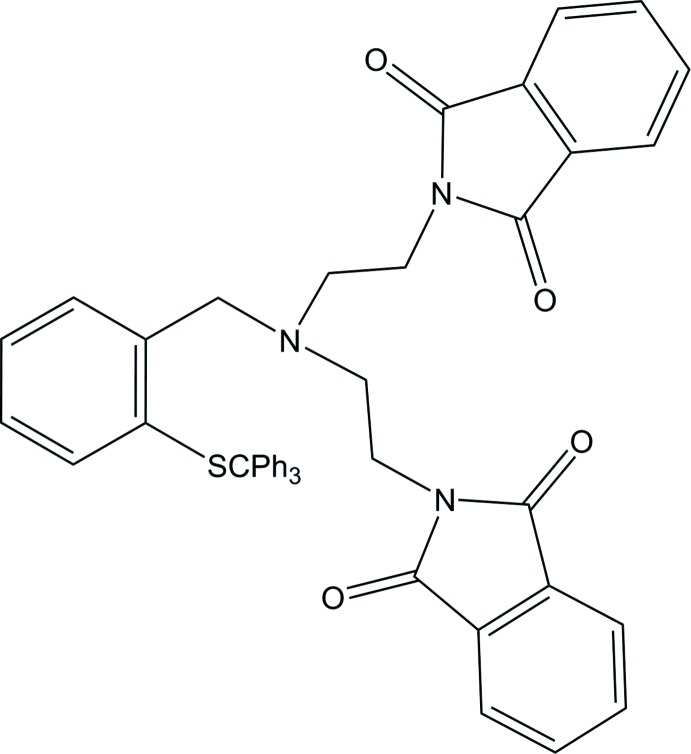


## Experimental   

### Crystal data  

C_46_H_37_N_3_O_4_S
*M* *_r_* = 727.85
Triclinic, 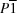

*a* = 9.8512 (11) Å
*b* = 11.5610 (13) Å
*c* = 16.945 (2) Å
α = 88.712 (3)°
β = 81.146 (3)°
γ = 69.421 (2)°
*V* = 1784.1 (4) Å^3^
*Z* = 2
Mo *K*α radiation
μ = 0.14 mm^−1^
*T* = 130 K
0.30 × 0.14 × 0.08 mm

### Data collection  

Bruker SMART APEX diffractometer
Absorption correction: multi-scan (*SADABS*; Sheldrick, 2004 ▶) *T*_min_ = 0.958, *T*_max_ = 0.989
17107 measured reflections
8464 independent reflections
6541 reflections with *I* > 2σ(*I*)
*R*_int_ = 0.029

### Refinement  

*R*[*F* ^2^ > 2σ(*F* ^2^)] = 0.046
*wR*(*F* ^2^) = 0.113
*S* = 1.02
8464 reflections
487 parameters
H-atom parameters constrained
Δρ_max_ = 0.32 e Å^−3^
Δρ_min_ = −0.26 e Å^−3^

### 

Data collection: *SMART* (Bruker, 2002 ▶); cell refinement: *SAINT* (Bruker, 2002 ▶); data reduction: *SAINT*; program(s) used to solve structure: *SHELXTL* (Sheldrick, 2008 ▶); program(s) used to refine structure: *SHELXTL*; molecular graphics: *SHELXTL*; software used to prepare material for publication: *SHELXTL* and local programs.

## Supplementary Material

Crystal structure: contains datablock(s) I. DOI: 10.1107/S1600536814015554/bt6987sup1.cif

Structure factors: contains datablock(s) I. DOI: 10.1107/S1600536814015554/bt6987Isup2.hkl

Supporting information file. DOI: 10.1107/S1600536814015554/bt6987Isup3.cml

**Fig. 1.**
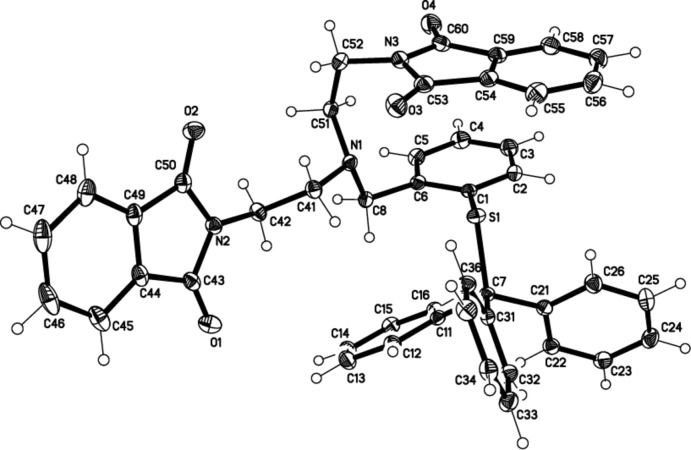
The molecular structure of the title compound. Displacement ellipsoids are drawn at the 50% probability level.

. DOI: 10.1107/S1600536814015554/bt6987fig1.tif
The mol­ecular structure of the title compound. Displacement ellipsoids are drawn at the 50% probability level.

CCDC reference: 1011916

Additional supporting information:  crystallographic information; 3D view; checkCIF report

## Figures and Tables

**Table 1:** Hydrogen-bond geometry (Å, °)

*D*—H⋯*A*	*D*—H	H⋯*A*	*D*⋯*A*	*D*—H⋯*A*
C33—H33*A*⋯O2^i^	0.95	2.55	3.194 (2)	125
C42—H42*A*⋯O1^ii^	0.99	2.54	3.378 (2)	142

Symmetry codes: (i) 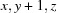; (ii) 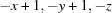.

## supplementary crystallographic information

### S1. Introduction

The development and synthesis of novel molecules with nitro­gen and sulfur as donor functions and studies of their metallation potential towards copper is important for the basic understanding of copper metalloproteins. In this context we have developed several tripodal thio­ether guanidine hybrids for modelling the active center of the peptidglycine-α-hy­droxy­lating monooxygenase (PHM) (Hoppe *et al.*, 2013; Neuba, 2009). The inter­mediate 3-(2-trityl­thio-benzyl)-1,5-diphthalimido-3-aza­pentan was obtained in a multi-step synthesis of tripodal thio­ether- and di­sulfide guanidine compounds.

### S2. Refinement

Crystal data, data collection and structure refinement details are summarized in Table 1. Hydrogen atoms were clearly identified in difference syntheses, refined at idealized positions riding on the carbon atoms with isotropic displacement parameters U_iso_(H) = 1.2U_eq_(C) and C–H 0.95–0.99 Å.

### S3. Preparation

The title compound was prepared as follows: to a suspension of bis­(phthalimido)di­ethyl­entriamin (10 mmol, 3.64 g)(Formica *et al.*, 2002), KI (0.5 mmol, 0.083 g) and K_2_CO_3_ (11.5 mmol,1.59 g) in 200 mL of dry MeCN a solution of (2-(bromo­methyl)­phenyl)(trityl)sulfane (10 mmol, 4.46 g) (Sagrera & Seoane, 2009) in dry MeCN (100 mL) was dropped over a period of 30 min. This mixture was then refluxed for 24 h. After the solvent was evaporated 200 ml water was added and the mixture was extracted with di­chloro­methane (3 x 80 mL). The combined organic layers were dried over Na_2_SO_4_. After filtration the solvent was removed und the crude product was obtained as a yellow oil. For purification, the raw product was stirred in Et_2_O (200 mL). The suspension was filtered and the collected solid dried under reduced pressure. Yield: 5.33 g (73 %). Yellow crystals suitable for X-ray diffraction were obtained by diffusion of Et_2_O into a cold saturated MeCN solution.

Spectroscopic data:

^1^H-NMR (500MHz, CDCl_3_, 25°C, δ [ppm]): 2.67 (t, 4H, CH_2_); 3.20 (s, 2H, CH_2_); 3.69 (t, 4H, CH_2_); 6.61 (dt, 1H, CH); 6.66 (dt, 1H, CH); 6.84 (dd, 1H,CH); 6.93 (dd, 1H, CH); 7.14-7.26 (m, 15H, CH); 7.68-7.69 (m, 4H, CH); 7.73-7.75 (m, 4H, CH).

^13^C-NMR (125MHz, CDCl_3_, 25°C, δ [ppm]): 35.6 (CH_2_); 51.2 (CH_2_); 55.5 (CH_2_); 70.8 (C_q_); 123.0 (CH); 126.3 (CH); 126.6 (CH); 127.5 (CH); 127.8 (CH); 129.3 (CH); 130.0 (CH); 132.4 (C_q_); 133.5 (CH); 133.6 (C_q_); 135.3 (CH); 143.9 (C_q_); 144.5 (C_q_); 168.1 (C_q_).

^15^N-NMR (50.7MHz, CDCl_3_, 25°C, δ [ppm]): 37.4 (N_Phimid_); 160.0 (N_tert_).

IR (KBr, [cm^-1^]): 3462br, 3053w, 2943w, 2814w, 2388w, 1774m, 1713s, 1616w, 1489w, 1468w, 1429m, 1394m, 1381m, 1356w, 1325w, 1275w, 1188w, 1103w, 1082m, 1028w, 1003w, 962w, 872w, 762w, 741w, 717m, 702m, 619w, 530w.

ESI-MS (m/z (%)): 727.9 (82) [M^+^]; 665.1 (4); 664.1 (8); 484.9 (5) [M^+^—CPh_3_]; 422.1 (6) [M^+^-Phimid]; 243.9 (20) [CPh_3_^+^]; 242.9 (100) [CPh_3_^+^-H].

Elemental analysis (*M* = 727.87 g mol^-1^): calcd. for C_46_H_37_N_3_O_4_S: C 75.91; H 5.12; N 5.77; S 4.41; O 8.79; found: C 75.75; H 5.38; N 5.79; S 4.52; O 8.56.

### Crystal data

**Table:**

C_46_H_37_N_3_O_4_S	*Z* = 2
*M**_r_* = 727.85	*F*(000) = 764
Triclinic, *P*1	*D*_x_ = 1.355 Mg m^−^^3^
Hall symbol: -P 1	Mo *K*α radiation, λ = 0.71073 Å
*a* = 9.8512 (11) Å	Cell parameters from 3271 reflections
*b* = 11.5610 (13) Å	θ = 2.2–25.6°
*c* = 16.945 (2) Å	µ = 0.14 mm^−^^1^
α = 88.712 (3)°	*T* = 130 K
β = 81.146 (3)°	Prism, yellow
γ = 69.421 (2)°	0.30 × 0.14 × 0.08 mm
*V* = 1784.1 (4) Å^3^	

### Data collection

**Table:**

Bruker SMART APEX diffractometer	8464 independent reflections
Radiation source: sealed tube	6541 reflections with *I* > 2σ(*I*)
Graphite monochromator	*R*_int_ = 0.029
φ and ω scans	θ_max_ = 27.9°, θ_min_ = 1.2°
Absorption correction: multi-scan (*SADABS*; Sheldrick, 2004)	*h* = −12→12
*T*_min_ = 0.958, *T*_max_ = 0.989	*k* = −15→14
17107 measured reflections	*l* = −22→22

### Refinement

**Table:**

Refinement on *F*^2^	Primary atom site location: structure-invariant direct methods
Least-squares matrix: full	Secondary atom site location: difference Fourier map
*R*[*F*^2^ > 2σ(*F*^2^)] = 0.046	Hydrogen site location: difference Fourier map
*wR*(*F*^2^) = 0.113	H-atom parameters constrained
*S* = 1.02	*w* = 1/[σ^2^(*F*_o_^2^) + (0.050*P*)^2^ + 0.3999*P*] where *P* = (*F*_o_^2^ + 2*F*_c_^2^)/3
8464 reflections	(Δ/σ)_max_ < 0.001
487 parameters	Δρ_max_ = 0.32 e Å^−^^3^
0 restraints	Δρ_min_ = −0.26 e Å^−^^3^

### Special details

**Table:**

Geometry. All e.s.d.'s (except the e.s.d. in the dihedral angle between two l.s. planes) are estimated using the full covariance matrix. The cell e.s.d.'s are taken into account individually in the estimation of e.s.d.'s in distances, angles and torsion angles; correlations between e.s.d.'s in cell parameters are only used when they are defined by crystal symmetry. An approximate (isotropic) treatment of cell e.s.d.'s is used for estimating e.s.d.'s involving l.s. planes.
Refinement. Refinement of *F*^2^ against ALL reflections. The weighted *R*-factor *wR* and goodness of fit *S* are based on *F*^2^, conventional *R*-factors *R* are based on *F*, with *F* set to zero for negative *F*^2^. The threshold expression of *F*^2^ > σ(*F*^2^) is used only for calculating *R*-factors(gt) *etc*. and is not relevant to the choice of reflections for refinement. *R*-factors based on *F*^2^ are statistically about twice as large as those based on *F*, and *R*- factors based on ALL data will be even larger.

### Fractional atomic coordinates and isotropic or equivalent isotropic displacement parameters (Å^2^)

**Table:**

	*x*	*y*	*z*	*U*_iso_*/*U*_eq_	
S1	0.33001 (5)	0.89677 (4)	0.31311 (2)	0.02056 (10)	
O1	0.65075 (14)	0.63074 (12)	−0.05262 (7)	0.0306 (3)	
O2	0.79795 (13)	0.33099 (11)	0.12875 (7)	0.0276 (3)	
O3	0.67682 (13)	0.60774 (12)	0.34739 (7)	0.0307 (3)	
O4	0.26290 (15)	0.53493 (12)	0.46184 (7)	0.0331 (3)	
N1	0.41323 (14)	0.61373 (12)	0.23802 (8)	0.0184 (3)	
N2	0.68997 (15)	0.49383 (12)	0.05046 (8)	0.0199 (3)	
N3	0.48163 (16)	0.54309 (13)	0.39344 (8)	0.0238 (3)	
C1	0.17103 (17)	0.85519 (14)	0.32807 (9)	0.0187 (3)	
C2	0.06309 (19)	0.90721 (15)	0.39377 (10)	0.0241 (4)	
H2A	0.0682	0.9731	0.4242	0.029*	
C3	−0.0517 (2)	0.86376 (17)	0.41512 (11)	0.0292 (4)	
H3A	−0.1242	0.8994	0.4602	0.035*	
C4	−0.06024 (19)	0.76844 (17)	0.37060 (11)	0.0278 (4)	
H4A	−0.1382	0.7379	0.3852	0.033*	
C5	0.04550 (18)	0.71761 (15)	0.30459 (10)	0.0233 (4)	
H5A	0.0378	0.6531	0.2738	0.028*	
C6	0.16271 (17)	0.75848 (14)	0.28215 (9)	0.0186 (3)	
C7	0.29721 (17)	1.03173 (14)	0.24520 (9)	0.0174 (3)	
C8	0.27856 (17)	0.69644 (14)	0.21187 (9)	0.0195 (3)	
H8A	0.2403	0.6485	0.1792	0.023*	
H8B	0.3010	0.7603	0.1780	0.023*	
C11	0.25935 (17)	0.99395 (14)	0.16782 (9)	0.0176 (3)	
C12	0.36525 (19)	0.95550 (15)	0.09935 (10)	0.0223 (3)	
H12A	0.4597	0.9600	0.0993	0.027*	
C13	0.3345 (2)	0.91094 (16)	0.03144 (10)	0.0263 (4)	
H13A	0.4080	0.8850	−0.0144	0.032*	
C14	0.1980 (2)	0.90401 (16)	0.02997 (10)	0.0262 (4)	
H14A	0.1774	0.8731	−0.0165	0.031*	
C15	0.09091 (19)	0.94280 (15)	0.09716 (10)	0.0233 (4)	
H15A	−0.0036	0.9388	0.0964	0.028*	
C16	0.12082 (17)	0.98723 (14)	0.16515 (9)	0.0198 (3)	
H16A	0.0465	1.0135	0.2106	0.024*	
C21	0.18301 (17)	1.15014 (14)	0.28775 (9)	0.0187 (3)	
C22	0.06990 (18)	1.23226 (15)	0.25225 (10)	0.0217 (3)	
H22A	0.0540	1.2114	0.2014	0.026*	
C23	−0.02033 (19)	1.34480 (16)	0.29038 (10)	0.0261 (4)	
H23A	−0.0986	1.3985	0.2659	0.031*	
C24	0.0030 (2)	1.37875 (16)	0.36312 (11)	0.0302 (4)	
H24A	−0.0576	1.4560	0.3885	0.036*	
C25	0.1161 (2)	1.29854 (17)	0.39888 (11)	0.0325 (4)	
H25A	0.1340	1.3214	0.4487	0.039*	
C26	0.20316 (19)	1.18524 (16)	0.36228 (10)	0.0256 (4)	
H26A	0.2781	1.1302	0.3884	0.031*	
C31	0.44613 (17)	1.05130 (14)	0.22988 (9)	0.0171 (3)	
C32	0.44891 (18)	1.16140 (15)	0.19578 (10)	0.0228 (4)	
H32A	0.3601	1.2219	0.1849	0.027*	
C33	0.57870 (18)	1.18405 (16)	0.17758 (10)	0.0245 (4)	
H33A	0.5783	1.2591	0.1534	0.029*	
C34	0.70925 (18)	1.09851 (16)	0.19416 (10)	0.0229 (4)	
H34A	0.7979	1.1153	0.1830	0.028*	
C35	0.70855 (18)	0.98849 (16)	0.22717 (10)	0.0249 (4)	
H35A	0.7977	0.9287	0.2382	0.030*	
C36	0.57920 (18)	0.96410 (15)	0.24439 (10)	0.0226 (4)	
H36A	0.5810	0.8873	0.2663	0.027*	
C41	0.54448 (17)	0.59701 (15)	0.18007 (9)	0.0209 (3)	
H41A	0.6309	0.5430	0.2029	0.025*	
H41B	0.5557	0.6783	0.1713	0.025*	
C42	0.54416 (17)	0.54034 (16)	0.09883 (9)	0.0210 (3)	
H42A	0.5063	0.4716	0.1080	0.025*	
H42B	0.4768	0.6038	0.0689	0.025*	
C43	0.72895 (19)	0.54141 (15)	−0.02272 (9)	0.0212 (3)	
C44	0.88069 (19)	0.45774 (16)	−0.05396 (10)	0.0232 (4)	
C45	0.9701 (2)	0.45992 (19)	−0.12490 (10)	0.0310 (4)	
H45A	0.9399	0.5229	−0.1621	0.037*	
C46	1.1067 (2)	0.3654 (2)	−0.13927 (11)	0.0371 (5)	
H46A	1.1703	0.3630	−0.1879	0.045*	
C47	1.1520 (2)	0.27468 (19)	−0.08416 (12)	0.0364 (5)	
H47A	1.2464	0.2122	−0.0956	0.044*	
C48	1.0620 (2)	0.27344 (17)	−0.01253 (12)	0.0296 (4)	
H48A	1.0928	0.2117	0.0255	0.036*	
C49	0.92563 (18)	0.36634 (15)	0.00068 (10)	0.0224 (4)	
C50	0.80346 (18)	0.38922 (15)	0.06896 (10)	0.0209 (3)	
C51	0.39970 (18)	0.50027 (14)	0.27165 (10)	0.0212 (3)	
H51A	0.4215	0.4377	0.2281	0.025*	
H51B	0.2975	0.5173	0.2983	0.025*	
C52	0.50462 (19)	0.44899 (15)	0.33200 (10)	0.0254 (4)	
H52A	0.4878	0.3760	0.3571	0.030*	
H52B	0.6073	0.4223	0.3042	0.030*	
C53	0.56558 (19)	0.61842 (16)	0.39327 (10)	0.0243 (4)	
C54	0.49004 (18)	0.71032 (15)	0.46086 (10)	0.0233 (4)	
C55	0.5294 (2)	0.80253 (16)	0.49059 (11)	0.0297 (4)	
H55A	0.6166	0.8162	0.4677	0.036*	
C56	0.4357 (2)	0.87450 (17)	0.55544 (11)	0.0322 (4)	
H56A	0.4599	0.9381	0.5777	0.039*	
C57	0.3078 (2)	0.85511 (17)	0.58821 (11)	0.0307 (4)	
H57A	0.2462	0.9057	0.6324	0.037*	
C58	0.2679 (2)	0.76328 (16)	0.55773 (10)	0.0272 (4)	
H58A	0.1796	0.7507	0.5795	0.033*	
C59	0.36278 (19)	0.69097 (16)	0.49412 (10)	0.0236 (4)	
C60	0.3549 (2)	0.58296 (16)	0.45108 (10)	0.0247 (4)	

### Atomic displacement parameters (Å^2^)

**Table:**

	*U*^11^	*U*^22^	*U*^33^	*U*^12^	*U*^13^	*U*^23^
S1	0.0216 (2)	0.0195 (2)	0.0227 (2)	−0.00858 (16)	−0.00733 (16)	0.00563 (15)
O1	0.0324 (7)	0.0352 (7)	0.0264 (6)	−0.0138 (6)	−0.0080 (5)	0.0081 (5)
O2	0.0271 (7)	0.0262 (6)	0.0298 (7)	−0.0109 (5)	−0.0027 (5)	0.0063 (5)
O3	0.0213 (6)	0.0357 (7)	0.0302 (7)	−0.0055 (6)	0.0001 (5)	−0.0024 (6)
O4	0.0376 (8)	0.0358 (8)	0.0289 (7)	−0.0192 (6)	0.0006 (6)	0.0007 (6)
N1	0.0164 (7)	0.0165 (7)	0.0200 (7)	−0.0045 (5)	0.0012 (5)	−0.0005 (5)
N2	0.0188 (7)	0.0216 (7)	0.0193 (7)	−0.0083 (6)	0.0001 (5)	−0.0003 (5)
N3	0.0252 (8)	0.0226 (7)	0.0201 (7)	−0.0047 (6)	−0.0022 (6)	0.0007 (6)
C1	0.0198 (8)	0.0171 (8)	0.0180 (8)	−0.0050 (6)	−0.0032 (6)	0.0033 (6)
C2	0.0290 (9)	0.0193 (8)	0.0202 (8)	−0.0051 (7)	−0.0005 (7)	−0.0004 (6)
C3	0.0264 (9)	0.0278 (9)	0.0250 (9)	−0.0030 (8)	0.0053 (7)	0.0013 (7)
C4	0.0194 (9)	0.0305 (10)	0.0318 (10)	−0.0090 (8)	0.0003 (7)	0.0049 (8)
C5	0.0201 (8)	0.0225 (8)	0.0269 (9)	−0.0074 (7)	−0.0027 (7)	−0.0001 (7)
C6	0.0159 (8)	0.0173 (8)	0.0199 (8)	−0.0026 (6)	−0.0026 (6)	0.0022 (6)
C7	0.0169 (8)	0.0158 (7)	0.0174 (7)	−0.0039 (6)	−0.0016 (6)	0.0021 (6)
C8	0.0202 (8)	0.0178 (8)	0.0196 (8)	−0.0058 (7)	−0.0019 (6)	−0.0011 (6)
C11	0.0184 (8)	0.0143 (7)	0.0191 (8)	−0.0040 (6)	−0.0046 (6)	0.0016 (6)
C12	0.0214 (8)	0.0241 (9)	0.0219 (8)	−0.0092 (7)	−0.0016 (7)	0.0003 (7)
C13	0.0285 (9)	0.0297 (9)	0.0201 (8)	−0.0108 (8)	0.0000 (7)	−0.0018 (7)
C14	0.0315 (10)	0.0272 (9)	0.0217 (8)	−0.0105 (8)	−0.0089 (7)	−0.0006 (7)
C15	0.0211 (8)	0.0229 (8)	0.0273 (9)	−0.0076 (7)	−0.0080 (7)	0.0023 (7)
C16	0.0181 (8)	0.0186 (8)	0.0203 (8)	−0.0034 (7)	−0.0032 (6)	0.0017 (6)
C21	0.0169 (8)	0.0197 (8)	0.0193 (8)	−0.0080 (6)	0.0012 (6)	0.0006 (6)
C22	0.0206 (8)	0.0220 (8)	0.0210 (8)	−0.0066 (7)	−0.0008 (7)	0.0013 (6)
C23	0.0214 (9)	0.0226 (9)	0.0284 (9)	−0.0033 (7)	0.0022 (7)	0.0033 (7)
C24	0.0300 (10)	0.0208 (9)	0.0325 (10)	−0.0050 (8)	0.0079 (8)	−0.0052 (7)
C25	0.0383 (11)	0.0317 (10)	0.0250 (9)	−0.0104 (9)	−0.0011 (8)	−0.0089 (8)
C26	0.0242 (9)	0.0257 (9)	0.0237 (9)	−0.0052 (7)	−0.0033 (7)	−0.0011 (7)
C31	0.0175 (8)	0.0182 (8)	0.0158 (7)	−0.0068 (6)	−0.0015 (6)	−0.0027 (6)
C32	0.0180 (8)	0.0200 (8)	0.0275 (9)	−0.0038 (7)	−0.0022 (7)	0.0017 (7)
C33	0.0227 (9)	0.0200 (8)	0.0288 (9)	−0.0074 (7)	0.0018 (7)	−0.0013 (7)
C34	0.0177 (8)	0.0283 (9)	0.0234 (8)	−0.0104 (7)	0.0013 (7)	−0.0050 (7)
C35	0.0165 (8)	0.0286 (9)	0.0258 (9)	−0.0033 (7)	−0.0032 (7)	0.0005 (7)
C36	0.0206 (8)	0.0211 (8)	0.0245 (8)	−0.0057 (7)	−0.0030 (7)	0.0032 (7)
C41	0.0177 (8)	0.0236 (8)	0.0206 (8)	−0.0075 (7)	0.0001 (6)	−0.0011 (6)
C42	0.0154 (8)	0.0259 (9)	0.0203 (8)	−0.0065 (7)	0.0000 (6)	−0.0007 (7)
C43	0.0248 (9)	0.0264 (9)	0.0171 (8)	−0.0146 (7)	−0.0028 (7)	−0.0017 (6)
C44	0.0245 (9)	0.0282 (9)	0.0214 (8)	−0.0160 (7)	0.0005 (7)	−0.0076 (7)
C45	0.0319 (10)	0.0464 (12)	0.0224 (9)	−0.0257 (9)	0.0027 (8)	−0.0073 (8)
C46	0.0286 (10)	0.0611 (14)	0.0283 (10)	−0.0285 (10)	0.0095 (8)	−0.0201 (9)
C47	0.0212 (9)	0.0427 (12)	0.0449 (12)	−0.0138 (9)	0.0059 (8)	−0.0231 (9)
C48	0.0233 (9)	0.0266 (9)	0.0387 (10)	−0.0095 (8)	−0.0008 (8)	−0.0112 (8)
C49	0.0209 (8)	0.0234 (8)	0.0250 (8)	−0.0120 (7)	0.0012 (7)	−0.0080 (7)
C50	0.0200 (8)	0.0212 (8)	0.0235 (8)	−0.0100 (7)	−0.0023 (7)	−0.0032 (7)
C51	0.0238 (9)	0.0174 (8)	0.0211 (8)	−0.0070 (7)	0.0002 (7)	−0.0015 (6)
C52	0.0285 (9)	0.0176 (8)	0.0246 (9)	−0.0022 (7)	−0.0020 (7)	−0.0004 (7)
C53	0.0218 (9)	0.0236 (9)	0.0239 (8)	−0.0027 (7)	−0.0063 (7)	0.0045 (7)
C54	0.0213 (9)	0.0210 (8)	0.0230 (8)	−0.0009 (7)	−0.0058 (7)	0.0030 (7)
C55	0.0244 (9)	0.0279 (10)	0.0354 (10)	−0.0062 (8)	−0.0073 (8)	0.0021 (8)
C56	0.0336 (10)	0.0246 (9)	0.0359 (10)	−0.0039 (8)	−0.0125 (8)	−0.0031 (8)
C57	0.0311 (10)	0.0274 (9)	0.0251 (9)	0.0008 (8)	−0.0053 (8)	−0.0026 (7)
C58	0.0266 (9)	0.0283 (9)	0.0228 (9)	−0.0056 (8)	−0.0032 (7)	0.0033 (7)
C59	0.0248 (9)	0.0231 (8)	0.0204 (8)	−0.0043 (7)	−0.0062 (7)	0.0045 (7)
C60	0.0277 (9)	0.0256 (9)	0.0190 (8)	−0.0071 (8)	−0.0037 (7)	0.0040 (7)

### Geometric parameters (Å, º)

**Table:**

S1—C1	1.7748 (17)	C24—H24A	0.9500
S1—C7	1.8820 (15)	C25—C26	1.385 (2)
O1—C43	1.206 (2)	C25—H25A	0.9500
O2—C50	1.2086 (19)	C26—H26A	0.9500
O3—C53	1.211 (2)	C31—C32	1.393 (2)
O4—C60	1.209 (2)	C31—C36	1.398 (2)
N1—C41	1.454 (2)	C32—C33	1.382 (2)
N1—C51	1.459 (2)	C32—H32A	0.9500
N1—C8	1.4632 (19)	C33—C34	1.384 (2)
N2—C50	1.397 (2)	C33—H33A	0.9500
N2—C43	1.400 (2)	C34—C35	1.380 (2)
N2—C42	1.462 (2)	C34—H34A	0.9500
N3—C53	1.396 (2)	C35—C36	1.387 (2)
N3—C60	1.402 (2)	C35—H35A	0.9500
N3—C52	1.457 (2)	C36—H36A	0.9500
C1—C2	1.396 (2)	C41—C42	1.539 (2)
C1—C6	1.409 (2)	C41—H41A	0.9900
C2—C3	1.388 (2)	C41—H41B	0.9900
C2—H2A	0.9500	C42—H42A	0.9900
C3—C4	1.383 (3)	C42—H42B	0.9900
C3—H3A	0.9500	C43—C44	1.488 (2)
C4—C5	1.386 (2)	C44—C45	1.382 (2)
C4—H4A	0.9500	C44—C49	1.387 (2)
C5—C6	1.393 (2)	C45—C46	1.393 (3)
C5—H5A	0.9500	C45—H45A	0.9500
C6—C8	1.510 (2)	C46—C47	1.388 (3)
C7—C11	1.530 (2)	C46—H46A	0.9500
C7—C21	1.540 (2)	C47—C48	1.391 (3)
C7—C31	1.544 (2)	C47—H47A	0.9500
C8—H8A	0.9900	C48—C49	1.383 (2)
C8—H8B	0.9900	C48—H48A	0.9500
C11—C12	1.399 (2)	C49—C50	1.489 (2)
C11—C16	1.402 (2)	C51—C52	1.527 (2)
C12—C13	1.388 (2)	C51—H51A	0.9900
C12—H12A	0.9500	C51—H51B	0.9900
C13—C14	1.379 (2)	C52—H52A	0.9900
C13—H13A	0.9500	C52—H52B	0.9900
C14—C15	1.390 (2)	C53—C54	1.494 (2)
C14—H14A	0.9500	C54—C59	1.383 (2)
C15—C16	1.384 (2)	C54—C55	1.384 (3)
C15—H15A	0.9500	C55—C56	1.393 (3)
C16—H16A	0.9500	C55—H55A	0.9500
C21—C22	1.393 (2)	C56—C57	1.387 (3)
C21—C26	1.399 (2)	C56—H56A	0.9500
C22—C23	1.396 (2)	C57—C58	1.389 (3)
C22—H22A	0.9500	C57—H57A	0.9500
C23—C24	1.378 (3)	C58—C59	1.384 (2)
C23—H23A	0.9500	C58—H58A	0.9500
C24—C25	1.388 (3)	C59—C60	1.491 (2)
			
C1—S1—C7	108.40 (7)	C32—C33—H33A	119.7
C41—N1—C51	115.26 (12)	C34—C33—H33A	119.7
C41—N1—C8	113.46 (12)	C35—C34—C33	118.92 (16)
C51—N1—C8	113.32 (13)	C35—C34—H34A	120.5
C50—N2—C43	111.89 (14)	C33—C34—H34A	120.5
C50—N2—C42	123.39 (13)	C34—C35—C36	120.78 (15)
C43—N2—C42	124.51 (13)	C34—C35—H35A	119.6
C53—N3—C60	112.16 (14)	C36—C35—H35A	119.6
C53—N3—C52	124.67 (14)	C35—C36—C31	120.73 (15)
C60—N3—C52	122.17 (15)	C35—C36—H36A	119.6
C2—C1—C6	119.76 (15)	C31—C36—H36A	119.6
C2—C1—S1	118.68 (13)	N1—C41—C42	114.38 (13)
C6—C1—S1	120.90 (12)	N1—C41—H41A	108.7
C3—C2—C1	120.76 (16)	C42—C41—H41A	108.7
C3—C2—H2A	119.6	N1—C41—H41B	108.7
C1—C2—H2A	119.6	C42—C41—H41B	108.7
C4—C3—C2	119.82 (16)	H41A—C41—H41B	107.6
C4—C3—H3A	120.1	N2—C42—C41	112.76 (13)
C2—C3—H3A	120.1	N2—C42—H42A	109.0
C3—C4—C5	119.66 (17)	C41—C42—H42A	109.0
C3—C4—H4A	120.2	N2—C42—H42B	109.0
C5—C4—H4A	120.2	C41—C42—H42B	109.0
C4—C5—C6	121.82 (16)	H42A—C42—H42B	107.8
C4—C5—H5A	119.1	O1—C43—N2	125.20 (16)
C6—C5—H5A	119.1	O1—C43—C44	129.16 (15)
C5—C6—C1	118.16 (15)	N2—C43—C44	105.62 (14)
C5—C6—C8	119.79 (15)	C45—C44—C49	121.46 (17)
C1—C6—C8	122.02 (14)	C45—C44—C43	129.94 (17)
C11—C7—C21	114.91 (12)	C49—C44—C43	108.58 (14)
C11—C7—C31	110.98 (12)	C44—C45—C46	116.91 (18)
C21—C7—C31	107.33 (13)	C44—C45—H45A	121.5
C11—C7—S1	107.99 (10)	C46—C45—H45A	121.5
C21—C7—S1	111.25 (10)	C47—C46—C45	121.52 (18)
C31—C7—S1	103.83 (10)	C47—C46—H46A	119.2
N1—C8—C6	111.43 (12)	C45—C46—H46A	119.2
N1—C8—H8A	109.3	C46—C47—C48	121.34 (18)
C6—C8—H8A	109.3	C46—C47—H47A	119.3
N1—C8—H8B	109.3	C48—C47—H47A	119.3
C6—C8—H8B	109.3	C49—C48—C47	116.79 (18)
H8A—C8—H8B	108.0	C49—C48—H48A	121.6
C12—C11—C16	117.76 (15)	C47—C48—H48A	121.6
C12—C11—C7	121.09 (14)	C48—C49—C44	121.97 (16)
C16—C11—C7	120.95 (14)	C48—C49—C50	130.19 (16)
C13—C12—C11	121.02 (16)	C44—C49—C50	107.83 (14)
C13—C12—H12A	119.5	O2—C50—N2	125.03 (15)
C11—C12—H12A	119.5	O2—C50—C49	128.90 (16)
C14—C13—C12	120.51 (16)	N2—C50—C49	106.07 (13)
C14—C13—H13A	119.7	N1—C51—C52	111.20 (14)
C12—C13—H13A	119.7	N1—C51—H51A	109.4
C13—C14—C15	119.30 (16)	C52—C51—H51A	109.4
C13—C14—H14A	120.3	N1—C51—H51B	109.4
C15—C14—H14A	120.3	C52—C51—H51B	109.4
C16—C15—C14	120.52 (16)	H51A—C51—H51B	108.0
C16—C15—H15A	119.7	N3—C52—C51	110.21 (13)
C14—C15—H15A	119.7	N3—C52—H52A	109.6
C15—C16—C11	120.87 (15)	C51—C52—H52A	109.6
C15—C16—H16A	119.6	N3—C52—H52B	109.6
C11—C16—H16A	119.6	C51—C52—H52B	109.6
C22—C21—C26	117.56 (15)	H52A—C52—H52B	108.1
C22—C21—C7	123.54 (14)	O3—C53—N3	125.67 (16)
C26—C21—C7	118.46 (14)	O3—C53—C54	128.85 (17)
C21—C22—C23	120.89 (16)	N3—C53—C54	105.47 (14)
C21—C22—H22A	119.6	C59—C54—C55	121.27 (17)
C23—C22—H22A	119.6	C59—C54—C53	108.43 (15)
C24—C23—C22	120.69 (16)	C55—C54—C53	130.30 (17)
C24—C23—H23A	119.7	C54—C55—C56	117.15 (18)
C22—C23—H23A	119.7	C54—C55—H55A	121.4
C23—C24—C25	119.11 (16)	C56—C55—H55A	121.4
C23—C24—H24A	120.4	C57—C56—C55	121.30 (18)
C25—C24—H24A	120.4	C57—C56—H56A	119.4
C26—C25—C24	120.32 (17)	C55—C56—H56A	119.4
C26—C25—H25A	119.8	C56—C57—C58	121.40 (17)
C24—C25—H25A	119.8	C56—C57—H57A	119.3
C25—C26—C21	121.38 (16)	C58—C57—H57A	119.3
C25—C26—H26A	119.3	C59—C58—C57	116.92 (17)
C21—C26—H26A	119.3	C59—C58—H58A	121.5
C32—C31—C36	117.78 (15)	C57—C58—H58A	121.5
C32—C31—C7	117.54 (13)	C54—C59—C58	121.95 (17)
C36—C31—C7	124.61 (14)	C54—C59—C60	108.18 (15)
C33—C32—C31	121.08 (15)	C58—C59—C60	129.85 (17)
C33—C32—H32A	119.5	O4—C60—N3	124.86 (16)
C31—C32—H32A	119.5	O4—C60—C59	129.52 (16)
C32—C33—C34	120.68 (16)	N3—C60—C59	105.61 (15)
			
C7—S1—C1—C2	−92.52 (13)	C8—N1—C41—C42	−60.72 (18)
C7—S1—C1—C6	96.80 (13)	C50—N2—C42—C41	70.20 (19)
C6—C1—C2—C3	0.8 (2)	C43—N2—C42—C41	−115.51 (17)
S1—C1—C2—C3	−169.98 (13)	N1—C41—C42—N2	−164.27 (13)
C1—C2—C3—C4	−0.5 (2)	C50—N2—C43—O1	179.60 (16)
C2—C3—C4—C5	−0.5 (3)	C42—N2—C43—O1	4.7 (3)
C3—C4—C5—C6	1.1 (3)	C50—N2—C43—C44	0.91 (18)
C4—C5—C6—C1	−0.7 (2)	C42—N2—C43—C44	−173.95 (14)
C4—C5—C6—C8	177.38 (14)	O1—C43—C44—C45	−0.6 (3)
C2—C1—C6—C5	−0.2 (2)	N2—C43—C44—C45	178.00 (17)
S1—C1—C6—C5	170.37 (12)	O1—C43—C44—C49	−179.04 (17)
C2—C1—C6—C8	−178.28 (14)	N2—C43—C44—C49	−0.42 (18)
S1—C1—C6—C8	−7.7 (2)	C49—C44—C45—C46	0.6 (3)
C1—S1—C7—C11	−56.76 (12)	C43—C44—C45—C46	−177.62 (17)
C1—S1—C7—C21	70.21 (13)	C44—C45—C46—C47	−1.3 (3)
C1—S1—C7—C31	−174.65 (10)	C45—C46—C47—C48	0.8 (3)
C41—N1—C8—C6	−153.09 (13)	C46—C47—C48—C49	0.3 (3)
C51—N1—C8—C6	72.99 (17)	C47—C48—C49—C44	−0.9 (3)
C5—C6—C8—N1	−103.61 (16)	C47—C48—C49—C50	178.11 (17)
C1—C6—C8—N1	74.43 (18)	C45—C44—C49—C48	0.5 (3)
C21—C7—C11—C12	135.76 (15)	C43—C44—C49—C48	179.05 (15)
C31—C7—C11—C12	13.77 (19)	C45—C44—C49—C50	−178.77 (15)
S1—C7—C11—C12	−99.42 (15)	C43—C44—C49—C50	−0.18 (18)
C21—C7—C11—C16	−49.43 (19)	C43—N2—C50—O2	179.79 (16)
C31—C7—C11—C16	−171.42 (13)	C42—N2—C50—O2	−5.3 (3)
S1—C7—C11—C16	75.40 (15)	C43—N2—C50—C49	−1.02 (18)
C16—C11—C12—C13	−0.7 (2)	C42—N2—C50—C49	173.91 (14)
C7—C11—C12—C13	174.26 (15)	C48—C49—C50—O2	0.7 (3)
C11—C12—C13—C14	0.2 (3)	C44—C49—C50—O2	179.87 (17)
C12—C13—C14—C15	0.3 (3)	C48—C49—C50—N2	−178.43 (17)
C13—C14—C15—C16	−0.4 (2)	C44—C49—C50—N2	0.72 (17)
C14—C15—C16—C11	−0.1 (2)	C41—N1—C51—C52	74.33 (17)
C12—C11—C16—C15	0.6 (2)	C8—N1—C51—C52	−152.62 (13)
C7—C11—C16—C15	−174.34 (14)	C53—N3—C52—C51	−97.34 (18)
C11—C7—C21—C22	−13.1 (2)	C60—N3—C52—C51	70.26 (19)
C31—C7—C21—C22	110.83 (17)	N1—C51—C52—N3	54.76 (18)
S1—C7—C21—C22	−136.22 (14)	C60—N3—C53—O3	−176.78 (15)
C11—C7—C21—C26	174.68 (14)	C52—N3—C53—O3	−8.1 (2)
C31—C7—C21—C26	−61.38 (18)	C60—N3—C53—C54	3.93 (17)
S1—C7—C21—C26	51.58 (18)	C52—N3—C53—C54	172.61 (13)
C26—C21—C22—C23	−0.5 (2)	O3—C53—C54—C59	177.07 (16)
C7—C21—C22—C23	−172.81 (15)	N3—C53—C54—C59	−3.68 (17)
C21—C22—C23—C24	1.8 (3)	O3—C53—C54—C55	−3.0 (3)
C22—C23—C24—C25	−1.1 (3)	N3—C53—C54—C55	176.26 (16)
C23—C24—C25—C26	−0.8 (3)	C59—C54—C55—C56	0.2 (2)
C24—C25—C26—C21	2.1 (3)	C53—C54—C55—C56	−179.73 (15)
C22—C21—C26—C25	−1.4 (3)	C54—C55—C56—C57	−0.6 (3)
C7—C21—C26—C25	171.32 (16)	C55—C56—C57—C58	0.0 (3)
C11—C7—C31—C32	77.93 (17)	C56—C57—C58—C59	1.0 (2)
C21—C7—C31—C32	−48.38 (18)	C55—C54—C59—C58	0.8 (2)
S1—C7—C31—C32	−166.28 (12)	C53—C54—C59—C58	−179.23 (14)
C11—C7—C31—C36	−98.98 (17)	C55—C54—C59—C60	−177.83 (15)
C21—C7—C31—C36	134.72 (15)	C53—C54—C59—C60	2.11 (17)
S1—C7—C31—C36	16.82 (18)	C57—C58—C59—C54	−1.4 (2)
C36—C31—C32—C33	−0.6 (2)	C57—C58—C59—C60	176.94 (16)
C7—C31—C32—C33	−177.69 (15)	C53—N3—C60—O4	178.27 (15)
C31—C32—C33—C34	−1.2 (3)	C52—N3—C60—O4	9.3 (2)
C32—C33—C34—C35	1.8 (3)	C53—N3—C60—C59	−2.70 (17)
C33—C34—C35—C36	−0.7 (3)	C52—N3—C60—C59	−171.71 (13)
C34—C35—C36—C31	−1.0 (3)	C54—C59—C60—O4	179.19 (17)
C32—C31—C36—C35	1.7 (2)	C58—C59—C60—O4	0.7 (3)
C7—C31—C36—C35	178.56 (15)	C54—C59—C60—N3	0.22 (17)
C51—N1—C41—C42	72.27 (17)	C58—C59—C60—N3	−178.30 (16)

### Hydrogen-bond geometry (Å, º)

**Table:**

*D*—H···*A*	*D*—H	H···*A*	*D*···*A*	*D*—H···*A*
C33—H33*A*···O2^i^	0.95	2.55	3.194 (2)	125
C42—H42*A*···O1^ii^	0.99	2.54	3.378 (2)	142

Symmetry codes: (i) *x*, *y*+1, *z*; (ii) −*x*+1, −*y*+1, −*z*.
