# Supplementary figures and images for: Crystal structure of 2,2′-({[2-(trityl­sulfan­yl)benz­yl]azane­diyl}bis­(ethane-2,1-di­yl))bis­(isoindoline-1,3-dione)
Source: Acta Crystallogr Sect E Struct Rep Online. 2014 Aug 1;70(Pt 9):o895–6. doi: 10.1107/S1600536814015554 (PMC4186125; doi:10.1107/S1600536814015554)

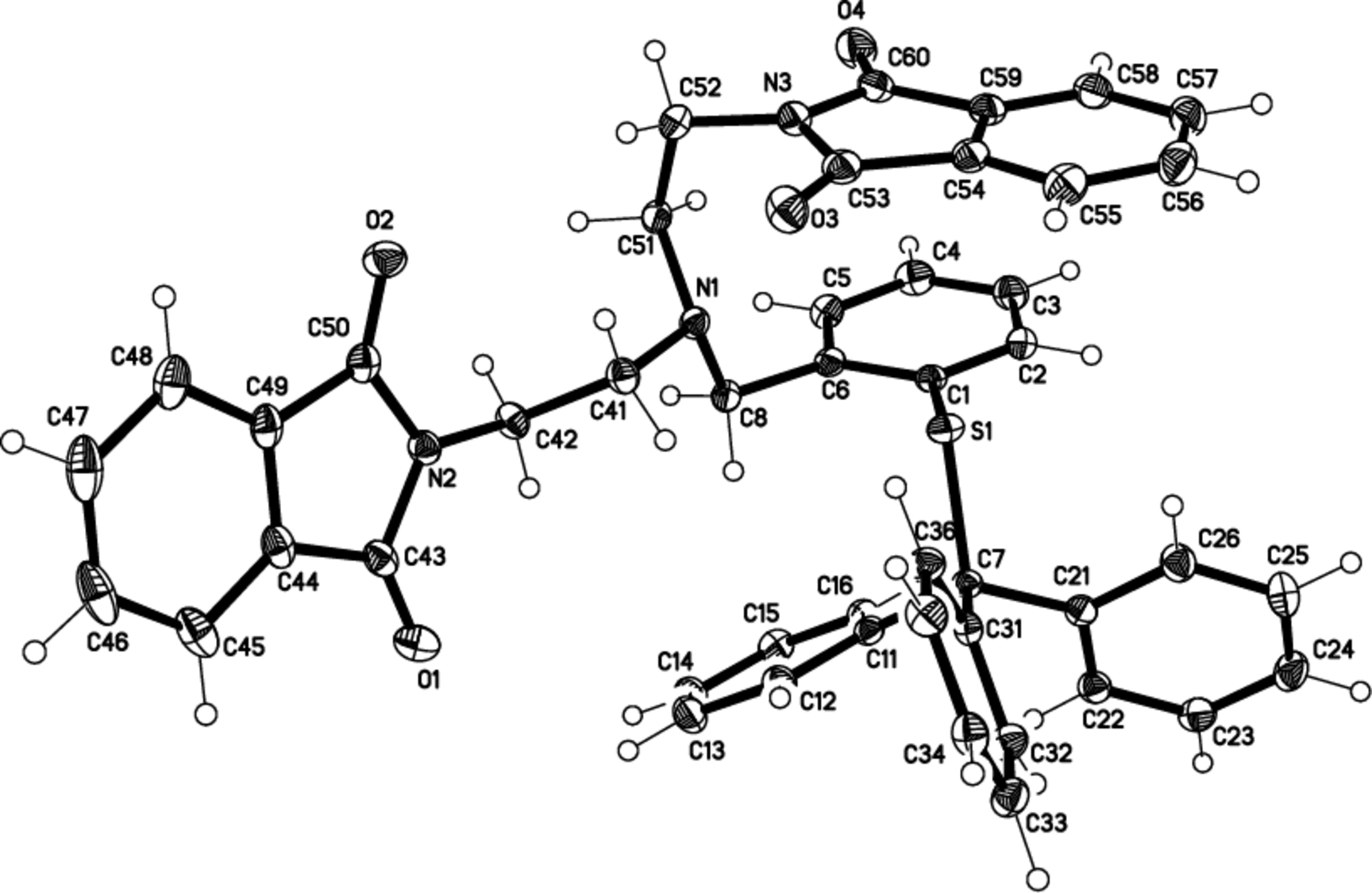

Supplement: Supplementary file 4 [file e-70-0o895-fig1.tif]
